# Atezolizumab and bevacizumab-induced encephalitis in advanced hepatocellular carcinoma: Case report and literature review

**DOI:** 10.1097/MD.0000000000026377

**Published:** 2021-06-18

**Authors:** Burcin Özdirik, Fabian Jost-Brinkmann, Lynn Jeanette Savic, Raphael Mohr, Frank Tacke, Christoph J. Ploner, Christoph Roderburg, Tobias Müller

**Affiliations:** a Department of Hepatology and Gastroenterology; b Department of Diagnostic and Interventional Radiology, Charité University Medicine Berlin; c Berlin Institute of Health (BIH); d Department of Neurology, Charité University Medicine Berlin, Berlin; e Clinic for Gastroenterology, Hepatology and Infectious Diseases, University Hospital Düsseldorf, Medical Faculty of Heinrich Heine University Düsseldorf, Düsseldorf, Germany.

**Keywords:** atezolizumab, bevacizumab, case report, encephalitis, hepatocellular carcinoma, immune checkpoint-inhibitor

## Abstract

**Introduction:**

On the basis of the results of the IMBRAVE-150 trial, the combination of atezolizumab, a programmed cell death ligand 1 (PD-L1) antibody, as well as bevacizumab, a *vascular endothelial growth factor* (VEGF) antibody, represents a promising novel first-line therapy in patients with advanced hepatocellular carcinoma (HCC). Despite favorable safety data, serious adverse events have been described. However, central nervous system complications such as encephalitis have rarely been reported. We present the case of a 70-year-old woman with hepatitis C virus (HCV)-related liver cirrhosis and advanced HCC who developed severe encephalitis after only one cycle of atezolizumab/bevacizumab.

**Patient concerns:**

Ten days after administration, the patient presented with confusion, somnolence, and emesis. Within a few days, the patient's condition deteriorated, and mechanical ventilation became necessary.

**Diagnosis:**

Cerebrospinal fluid (CSF) analysis showed increased cell count and elevated protein values. Further work-up revealed no signs of an infectious, paraneoplastic, or other autoimmune cause.

**Intervention:**

Suspecting an atezolizumab/bevacizumab-related encephalitis, we initiated a high-dose steroid pulse therapy as well as repeated plasmapheresis, which resulted in clinical improvement and remission of CSF abnormalities.

**Outcome:**

Despite successful weaning and transfer to a rehabilitation ward, the patient died of progressive liver cancer 76 days after initial treatment with atezolizumab/bevacizumab, showing no response.

**Conclusion:**

This case illustrates that rapid immunosuppressive treatment with prednisolone can result in remission even of severe encephalitis. We discuss this case in the context of available literature and previously reported cases of atezolizumab-induced encephalitis in different tumor entities, highlighting the diagnostic challenges in oncologic patients treated with immune checkpoint-inhibitors.

## 1 Introduction

Hepatocellular carcinoma (HCC) is the most frequent primary liver cancer, representing the fifth most common cancer worldwide.^[1,2]^ The development of HCC is associated with chronic liver disease in up to 90% of cases.^[2]^ The most frequent risk factors include chronic viral hepatitis (types B and C), alcohol intake, metabolic disease, and aflatoxin exposure.^[3]^ HCCs are commonly classified according to the Barcelona Clinic of Liver Cancer (BCLC) staging system, which stratifies HCC into 5 different classes (BCLC 0 and A–D) based on the degree of liver dysfunction, tumor burden, and performance status.^[2,3]^ While in patients with early tumor stages (BCLC A, B), surgical resection, orthotopic liver transplantation, or ablative therapies might offer a curative approach, approximately 50% of patients are diagnosed with locally advanced or metastatic disease and, therefore, are not eligible for these potentially curative treatments.^[2,4]^ In case of advanced disease, systemic therapy is recommended by current guidelines.^[4]^

For the last decade, sorafenib, a multireceptor tyrosine-kinase inhibitor targeting predominantly vascular endothelial growth factor receptor (VEGFR) 2 and Raf kinase inhibition was considered standard of care for first-line systemic treatment of HCC.^[5,6]^ On the basis of the results of the REFLECT-trial using a non-inferiority endpoint design, lenvatinib, another multireceptor tyrosine-kinase inhibitor, was recently approved as an alternative to sorafenib for first-line therapy.^[7]^ The rather poor efficacy but important toxic profile of both agents led to further studies, mainly involving immunotherapies with immune checkpoint inhibitors (ICIs). In this context, the multicenter phase III study IMBRAVE 150 compared the efficacy of a combination of atezolizumab as well as bevacizumab with sorafenib in patients with advanced HCC. The combination therapy could improve all oncological endpoints (overall survival, progression-free survival, overall response rate). In addition, the combination of atezolizumab and bevacizumab was significantly less toxic compared with sorafenib.^[8]^

The most common side effects of this combination were loss of appetite, hypertension, fatigue, rash, and gastrointestinal symptoms. Nevertheless, treatment with ICI may entail immune-related adverse events (irAE) by creating an exaggerated inflammatory response, as blocking the T-cell inhibitory programmed cell death ligand 1 (PD-L1) pathway carries the risk of inducing or exaggerating autoimmune diseases. Such irAEs include dermatological, gastrointestinal, hepatological, endocrine, or rheumatological toxicities as well as pneumonitis.^[9]^ Up to now, only few case reports and small case series have reported central nervous system complications such as encephalitis and encephalopathy.^[10–14]^

Here, we report one of the first cases of severe encephalitis after treatment with atezolizumab and bevacizumab for advanced HCC that was successfully treated by high-dose steroids, plasmapheresis, and drug discontinuation.

## 2 Case presentation

**Figure 1:**
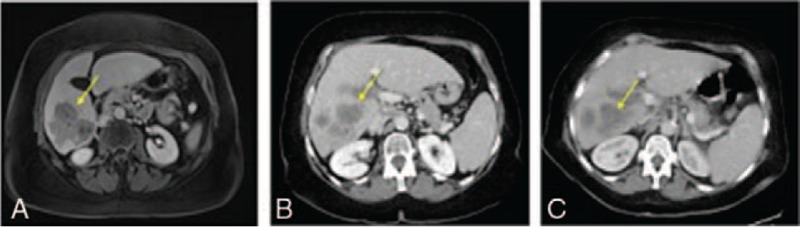
Multifocal hepatocellular carcinoma with macrovascular invasion into the right portal vein in axial abdominal CT- and MR-imaging during the course of treatment. Tumor lesions are indicated by yellow arrows. (A) Axial contrast-enhanced magnetic resonance imaging (MRI) (venous phase) demonstrates multifocal hepatocellular carcinoma with macrovascular invasion into the right portal vein at initial diagnosis in March 2020. (B) Axial contrast-enhanced computed tomography (CT)-scan (venous phase) displays a progression in size 1 week after initial diagnosis. (C) Axial contrast-enhanced CT-scan (venous phase) displays a progression in size of multifocal hepatocellular carcinoma 2 weeks after 1 cycle of atezolizumab/bevacizumab.

A 70-year-old female patient without pre-existing conditions was diagnosed with multifocal hepatocellular carcinoma with macrovascular invasion into the right portal vein in March 2020. Routine ultrasound examination of her hepatitis C induced liver cirrhosis revealed multiple HCC foci in segments V, VI, VII, and I (Fig. 1).

Further clinical work-up, including magnetic resonance imaging (MRI) and computer tomography (CT)-scan of the chest, did not reveal extrahepatic tumor manifestation except lymph node invasion (portocaval, paraaortal). Liver function was well preserved [Child-Pugh-Score A, model of End-stage liver disease (MELD) 6]; therefore, a tumor stage of BCLC C was assigned. Serum level of alfa-fetoprotein (AFP) was 430 U/mL. As results from the IMBRAVE-150 trial showed superiority over sorafenib with regard to progression-free survival and tumor response, we initiated treatment with atezolizumab (fix dose of 1200 mg over 2 hours) and bevacizumab (15 mg/kg over 90 minutes).^[15]^ Initial administration was well tolerated without any signs of acute toxicity.

Ten days later, the patient presented herself in our emergency room with previous episodes of impaired cognition and language, somnolence, emesis, and dyspnea, along with severe deterioration of her general condition. Neurological examination at initial presentation did not show any abnormalities except for a blurred orientation to time. Laboratory testing revealed slightly elevated inflammatory markers [C-reactive protein (CRP) 54 mg/dL (<5 mg/dL), leucocytes 3.08/nL (4–10/nL), lymphocytes 0.34/nL (1.10–4.50/nL), sodium 130 mmol/L (135–145 mmol/L), thyroid-stimulating hormone (TSH) 0.20 mU/L (0.27–4.20 mU/L), fT3 1.79 U/L (2–4.40 U/L), ALT 61 U/l (<31 U/L), AST 92 U/L (<35 U/L)]. No clinical or laboratory signs of hepatic encephalopathy were present [ammonia 31 μmol/L (21–71 μmol/L)]. Blood, urine, and sputum cultures did not identify any pathogen. COVID 19 and influenza A and B were negative. A cranial CT-scan did not show any signs of bleeding or ischemia. Chest x-ray did not reveal any pathological findings.

In this clinical setting, we initiated treatment with methylprednisolone 100 mg/day (1 mg/kg) accompanied by an anti-infective therapy regime with ceftriaxone, amoxicillin, and acyclovir as long as the laboratory results were in progress. We initially suspected atezolizumab-related hypophysitis due to low TSH and fT3 levels combined with progressive somnolence and hyponatremia. Further clinical work-up, including cranial MRI with gadobutrol, did not reveal any abnormal findings, displaying no signs of cerebral metastases, bleeding, ischemia, or inflammation with particular regard to the pituitary gland. Moreover, initial endocrine tests, including cortisol, Insulin-like growth factor 1, luteinizing hormone, follicle-stimulating hormone, and adrenocorticotropic hormone levels were unremarkable, displaying no signs of hypophysitis.

The clinical condition of the patient deteriorated with recurrent fever episodes, adynamia, and somnolence. Cerebral spinal fluid (CSF) analysis on day 4 (3 days after treatment initiation with methylprednisolone) revealed an elevated leucocyte count (179/μL) and increased protein level (5494 mg/dL) (Table 1).

**Table 1:** Results of cerebrospinal fluid analysis during high-dose prednisolone treatment.

Days after initial presentation, d	1	2	3	4–7	8	9–12	13	14–21	22–27	32–37	38–45
Prednisolone, mg	100	100	100	150	150	1000	1000	60	50	40	30
Plasmapheresis				Session 1,2,3							
Cell count, cells/μL			179		34		4				
Protein level, mg/L			5494		2097		825				
Lactate, mg/dL			56		43		32				
Glucose, mg/dL			71		72		64				

CSF cytology showed no tumor cells. Further CSF work-up showed no signs of an infectious (bacterial, viral, or fungal), autoimmune or paraneoplastic inflammation of the central nervous system, rendering an atezolizumab-associated encephalitis likely (detailed analysis is listed in Table 2).

**Table 2:** Cerebrospinal fluid and serum analysis results.

Infectious parameters	Result	Paraneoplastic and autoimmune parameters	Result
Herpes simplex-1/2 DNA	Negative	Voltage-gated-potassium antibody	Negative
Human herpesvirus-6 DNA	Negative	Anti-Hu antibody	Negative
Varicella-zoster-virus IgM, G	Negative	Anti-Yo antibody	Negative
Cytomegalovirus DNA	Negative	Anti-Ri antibody	Negative
Adenovirus DNA	Negative	*N*-methyl-d-aspartate antibody	Negative
Enterovirus RNA	Negative	Amphyphysin antibody	Negative
John Cunnigham virus DNA	Negative	Anti-CV2 antibody	Negative
Cryptococcal antigen	Negative	Aquaporin 4 antibody	Negative
Rickettsia IgG/IgM	Negative	Contactin-associated-protein-like-2 antibody	Negative
*Ehrlichia chaffeensis* DNA	Negative	DPP-like protein 6 antibody	Negative
*Borrellia burgdorferi* Ig M, G	Negative	Dopamin-2-antibody	Negative
*Treponoma pallidum* Ig M, G	Negative	GAD65 antibody	Negative
*E coli* DNA	Negative	GABA-B-receptors	Negative
Listeria DNA	Negative	AMPAR1 and 2 antibodies	Negative
Neisseria DNA	Negative	LGl1 antibody	Negative
*Streptococcus aglatiae* DNA	Negative	Myelin antibodies	Negative
*Streptococcus pneumonia* DNA	Negative		

On day 5, the patient's general condition deteriorated with progressive respiratory failure. A subsequent CT-scan revealed bilateral infiltrations of the lung and pleural effusions, indicating aspiration pneumonia. Thus, the patient was admitted to the intensive care unit, where she was mechanically ventilated. Anti-infective therapy was escalated to piperacillin and tazobactam. Subsequently, we decided to start treatment with plasmapheresis, following the recommendations of the guidelines for ICI-associated adverse events (AEs) by the American society of clinical oncology.^[16]^ After 3 sessions of plasmaphereses, serum levels of Creatine-kinase (CK) and myoglobin (initially CK 1700 U/L, myoglobin 1600 U/L) returned to normal. Moreover, the dose of methylprednisolone was increased up to 150 mg per day (1.5 mg/kg/day) and 2 days later up to 200 mg/day (2 mg/kg/day).

**Figure 2:**
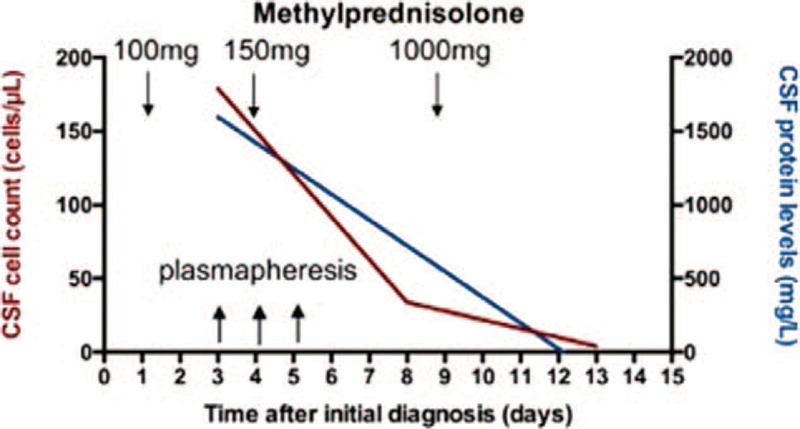
Cerebrospinal fluid (CSF) cell count and protein levels during treatment with prednisolone and plasmapheresis. The graph displays CSF cell count and protein levels during course of treatment with prednisolone and plasmapheresis. Initially, prednisolone 100 mg/day (1 mg/kg) was administered intravenously and later increased up to 150 mg/day intravenously. When CSF analysis on day 8 did not reveal liquor remission, the dose was increased up to 1000 mg/day. Complete liquor remission was achieved on day 13.

On day 8, CSF analysis revealed a decrease in cell count and protein level. The patient's cognition was still severely impaired and she was even unable to follow simple instructions. Thus, steroid pulse therapy with 1 g methylprednisolone for 5 days was initiated, leading to further remission of clinical symptoms (patient could follow easy instructions) and CSF cell count (Table 1, Fig. 2).

Neurological examination still revealed areflexia and a reduced tone in all 4 extremities. Electromyography (EMG) on day 10 demonstrated a motor neuropathy with axonal and proximal demyelinating component, suggesting a critical illness myopathy possibly accompanied by immune-mediated polyradiculoneuropathy.

In the following days, a CT-scan of the chest indicated a resolution of infiltrations parallel to clinical improvement. Nevertheless, several weaning attempts failed, probably due to persistent muscle weakness. Therefore, we performed dilatative tracheostomy on day 15. Afterwards, communication with the patient improved considerably. She started to follow commands correctly, still not being able to mobilize on her own. Motor examination of the extremities revealed a decreased tone, proximal and distal muscle weakness, and areflexia. Twenty-one days days after initial presentation, the patient was transferred to a weaning rehabilitation clinic. Decannulation could be performed after 42 days when sufficient spontaneous breathing was possible. After 2 months, the patient started to masticate and swallow. Active mobilization was still not possible, as she still suffered from severe critical illness neuropathy. At this time-point, the patient's general condition deteriorated again due to progressive liver cancer disease. After discontinuation of atezolizumab/bevacizumab treatment after initial administration and under consideration of the patient's will and her poor general condition, no new HCC treatment approaches were initiated. Seventy-six days after initial treatment with atezolizumab/bevacizumab, the patient died due to multiorgan failure.

## 3 Discussion and conclusion

ICIs are a mainstay in the treatment of many cancers including HCC. The principle of ICI treatment is to overcome immune tolerance against the tumor, which is mediated by specific inhibitory molecules such as PD-1, the PD-1 ligand (PDL-1), or cytotoxic T-lymphocyte-associated Protein 4 (CTLA-4). Once activated, they repress the T-cell response against cancer cells. In turn, antibodies against these immune checkpoints might activate the immune system to re-recognize these tumor cells as foreign.^[17]^ Atezolizumab blocks the PD-1/PD-L1 signaling by selectively binding to PD-L1, enabling the inhibition of effector T-cells with induction of tumor cell death.^[18,19]^ Nevertheless, despite a well-tolerability and manageable safety profile, ICI treatment goes along with a risk for irAEs. In a Ib study on the toxicity of atezolizumab and bevacizumab in advanced HCC, Lee et al reported 12 patients (12%) who required systemic corticosteroids within 30 days of an irAE with atezolizumab. These patients developed pneumonitis, colitis, drug-induced liver injury, and an increase in liver enzymes.^[20]^ Moreover, the expert group around Pishvaian et al even described a case of encephalitis, but did not report further clinical details.^[21]^ Besides this case, to our knowledge, only a few cases of encephalitis following treatment with atezolizumab have been published (Table 3).

**Table 3:** Summary of characteristics of previously reported atezolizumab-induced encephalitis cases.

	Sex, age	Primary tumor	Agent, dose	Time to onset	Initial symptoms	CSF	cMRI	Treatment	Outcome
^[10]^	F, 59	bladder	Atezolizumab1200 mg	12 days	Confusion, fatigue, spastic tremors, emesis	Increased cell count and protein levels	Isolated frontal lobe metastasis, no other pathological findings	Dexamethasone 40 mg/day (10 mg every 6 h)	Residual upper extremity weakness 4/5; death after 1 month after discharge due to progressive disease
^[11]^	M, 78	lung	Atezolizumab1200 mg	13 days	Confusion, fever, somnolence, nuchal rigidity	Increased cell count and protein levels. On day 22 remission in CSF	No pathological findings	1 g methyl-prednisolone	Discharge after 58 days; well orientated, able to communicate and to sit down
^[12]^	F, 53	cervix	Atezolizumab 1200 mg; bevacizumab (15 mg/kg)	13 days	Altered mental, status headache, meningeal signs	Increased cell count, protein and glucose levels	Diffuse leptomeningeal enhancement	Dexamethasone 18 mg/day (6 mg every 8 h)	Discharge after 35 days to hospice; well-orientated, able to sit and communicate. Motor examination showed decreased tone, proximal and distal muscle weakness, preserved reflexes
^[13]^	F, 48	lung	Atezolizumab 1200 mg	13 days	Fever, psychomotor slow-down, memory impairment, aphasia	Increased cell count, elevated protein and glucose level	Pachy- and leptomeningeal enhancement	Methylprednisolone 1 g/d for 3 days, then 1 mg/kg per day for 1 month followed by a very gradual decrease	Complete resolution of neurological symptoms and brain MRI abnormalities after 9 mo
^[14]^	M, 49	bladder	Atezolizumab 1200 mg	N.a.	Altered mental status, stupor, generalized tonic-clonic seizures	Increased cell count	Diffuse leptomeningeal enhancement	Dexamethasone, intravenous immunoglobulin	Death due to septic shock and multiorgan failure

In summary, including our patient, the majority of patients were female (n = 4; 67%) with a mean age of 64 years (range 48–78). Primary tumor localizations were the lung (n = 2), bladder (n = 2), and the cervix (n = 1). Similar to our case, patients presented within 10 to 13 days after treatment initiation with altered mental status, confusion, and in 2 cases with meningeal signs. CSF cell count and protein levels were elevated in all patients during the course of disease, while no signs of an infectious, autoimmune, or paraneoplastic cause could be found (Table 2). In 3 reported cases, leptomeningeal enhancement was detected in MRI.^[12–14]^ According to Laserna et al,^[12]^ these findings were associated with a prolonged clinical course. In our case, pathological findings in imaging were absent. Moreover, duration of neurological alterations was longer than in previously reported cases which may be influenced by multiple factors: First, our patient developed aspiration pneumonia that required mechanical ventilation, which led to enhanced muscle weakness and prolonged weaning duration. Second, in line with the case reported by Laserna et al,^[12]^ our patient most likely suffered from critical illness myopathy and immune-mediated polyradiculoneuropathy as indicated by clinical findings (weakness, partial areflexia, reduced tone) and EMG analysis. Third, as bevacizumab further enhances atezolizumab's efficacy by reversing VEGF-mediated immunosuppression to promote T-cell infiltration into the tumor, more severe AEs in combination with bevacizumab compared with atezolizumab monotherapy can be assumed. In line with Kim et al,^[22]^ in our case, a rapid clinical improvement started approximately 26 days after atezolizumab/bevacizumab administration, which is plausible as the half-life of atezolizumab is 27 days.

Treatment of serious irAEs includes stopping the ICI agent and temporary immunosuppression with steroids or other agents such as intravenous immunoglobulins, plasmapheresis, or even infliximab or rituximab. The former might suppress autoimmune T-cell activity and the latter might help neutralizing the ICIs.^[16,23]^ In 50% of the reported cases, steroid dose up to 1 g/day was administered. In line, as no immediate clinical and CSF improvement was achieved, we escalated our therapy regime up to 1 g/day. In contrast to the reported cases, we started treatment with plasmapheresis for 3 days. As simultaneous steroid pulse therapy was performed, it is difficult to evaluate the therapeutic effect of plasmapheresis. However, CK and myoglobin levels decreased drastically. Therefore, in line with Möhn et al,^[24]^ we suggest a low threshold for therapy options such as intravenous immunoglobulins or plasmapheresis, as encephalitis is a highly relevant and serious complication. Consequently, the establishment of specific algorithms incorporating predictors is highly desirable in order to select optimal treatment strategies with regard to dosage and agents (steroid, infliximab, IVIG, rituximab, plasmapheresis) of immunosuppressive therapy.

To prevent irAE, it is important to identify risk factors for the development. Patients with pre-existing neurological autoimmune disorders probably represent a high-risk group. Garcia et al reported about 14 patients (including 8 patients with a previous history of multiple sclerosis) developing multiple sclerosis after treatment with pembrolizumab and nivolumab. All patients presented with rapid neurological disease progression and required medical intervention.^[24,25]^ In contrast, none of the patients known to us (neither our case patient nor the previously reported patients) suffered from a neurological disease before the initiation of treatment.

Another important aspect is the question, whether the occurrence of irAE correlates with the antitumor effect of ICI therapy. Our patient presented with further tumor progression 2 weeks after 1 cycle of atezolizumab and bevacizumab (Fig. 1). As treatment was immediately discontinued in all reported cases, it is difficult to evaluate treatment response. Nevertheless, in none of the reported cases, a remarkable treatment response was demonstrated. Therefore, future research should explore further risk factors to reduce irAE and to maximize the benefit of immunotherapy among patients.

## 4 Conclusion

We describe the clinical course of a patient with advanced HCC developing life-threatening encephalitis after only 1 cycle of atezolizumab and bevacizumab, which resulted in remission after rapid immunosuppressive treatment. In this regard, establishment of specific algorithms incorporating predictors is highly desirable in order to select optimal treatment strategies of immunosuppressive therapy with regard to dosage and agents (steroid, infliximab, IVIG, rituximab, plasmapheresis). Moreover, there is an unmet need for clinical trials to identify risk factors for developing irAE after atezolizumab and bevacizumab treatment in patients with HCC as well as preventive measures in respect to toxicity of this efficacious combination therapy.

## Author contributions

**Conceptualization:** Burcin Özdirik, Fabian Jost-Brinkmann, Frank Tacke, Christoph J. Ploner, Tobias Müller.

**Data curation:** Burcin Özdirik, Fabian Jost-Brinkmann.

**Supervision:** Christoph J. Ploner, Christoph Roderburg, Tobias Müller.

**Visualization:** Lynn Jeanette Savic.

**Writing – original draft:** Burcin Özdirik, Fabian Jost-Brinkmann, Tobias Müller.

**Writing – review & editing:** Burcin Özdirik, Fabian Jost-Brinkmann, Lynn Jeanette Savic, Raphael Mohr, Frank Tacke, Christoph J. Ploner, Christoph Roderburg, Tobias Müller.
